# Enhanced Thermal‐ and Photostability of Trace Pyrazine‐Incorporated Hydrogen Boride Nanosheets

**DOI:** 10.1002/smll.202506230

**Published:** 2025-10-22

**Authors:** Miwa Hikichi, Jumpei Takeshita, Junyan Han, Shin‐ichi Ito, Osamu Oki, Ryuki Tsuji, Akira Hasegawa, Samuel Jeong, Yoshikazu Ito, Iwao Matsuda, Hayato Tsurugi, Masahiro Miyauchi, Takahiro Kondo

**Affiliations:** ^1^ Department of Materials Science Institute of Pure and Applied Sciences University of Tsukuba Tsukuba Ibaraki 305‐8573 Japan; ^2^ Department of Applied Physics Institute of Pure and Applied Sciences University of Tsukuba Tsukuba Ibaraki 305‐8573 Japan; ^3^ Department of Materials Science and Engineering School of Materials and Chemical Technology Institute of Science Tokyo Meguro‐ku Tokyo 152‐8552 Japan; ^4^ Institute for Solid State Physics The University of Tokyo Kashiwa Chiba 277‐8581 Japan; ^5^ Department of Applied Chemistry Graduate School of Engineering The University of Osaka Suita Osaka 565‐0871 Japan; ^6^ Hydrogen Boride Research Center Tsukuba Institute for Advanced Research (TIAR) University of Tsukuba Tsukuba Ibaraki 305‐8577 Japan; ^7^ The Advanced Institute for Materials Research Tohoku University Sendai Miyagi 980‐8577 Japan

**Keywords:** hydrogen boride nanosheets, hydrogen release, molecular incorporation, photostability, pyrazine, stabilization, thermal stability

## Abstract

Hydrogen boride (HB) nanosheets are emerging hydrogen‐rich materials with controllable hydrogen‐release properties under UV, electrochemical, and thermal stimuli. However, their practical application in high‐temperature environments and harsh conditions, such as neutron shielding, requires improved stability against heat and light exposure. Herein, the synthesis of pyrazine‐incorporated HB nanosheets (Pyrazine‐HB) is reported via a simple solution‐mixing and drying process. Despite a low pyrazine content of ≈2.9 mol%, Pyrazine‐HB exhibits significantly enhanced thermal stability, with a hydrogen release temperature ≈200 K higher than that of pure HB. Moreover, its hydrogen release under UV and visible light irradiation is markedly suppressed. Brunauer–Emmett–Teller analysis reveals an increased surface area upon pyrazine incorporation, suggesting intercalation between HB layers. This is further supported by transmission electron microscopy, which showed changes in interlayer spacing indicative of molecular intercalation. The improved stability of Pyrazine‐HB is attributed to expanded interlayer spacing, which prevents interlayer hydrogen recombination. This study presents a facile and effective molecular‐level strategy for tuning the thermal stability and photostability of HB nanosheets, thereby advancing their potential for energy‐related applications.

## Graphical abstract

Pyrazine‐incorporated hydrogen boride (Pyrazine‐HB) nanosheets exhibit significantly enhanced thermal and photostability while retaining a high hydrogen content. Trace pyrazine incorporation increases the hydrogen release temperature by ≈200 K and suppresses UV‐triggered desorption. This study demonstrates a facile molecular incorporation strategy for stabilizing HB nanosheets and advancing their applicability in energy‐storage environments.

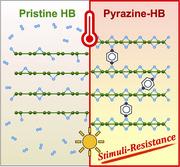

## 1 Introduction

Recent advances in 2D materials have highlighted their potential for energy‐related applications, including catalysis.^[^ ^1^, ^2^, ^3^ ^]^ Among them, boron‐based 2D materials such as borophene have attracted considerable attention for hydrogen storage, catalysis, and sensing, owing to their unique structural motifs and chemical versatility.^[^ ^4^, ^5^, ^6^, ^7^, ^8^, ^9^ ^]^ Recent studies have also emphasized the importance of interfacial regulation—through functional group engineering,^[^ ^10^, ^11^, ^12^ ^]^ heteroatom doping,^[^ ^13^, ^14^, ^15^ ^]^ and substrate selection^[^ ^16^ ^]^—in tuning the stability and reactivity of such layered systems.

Hydrogen boride (HB) nanosheets, commonly referred to as hydrogenated borophene or borophane, have emerged as promising solid‐state hydrogen carriers, offering a high gravimetric hydrogen capacity of 8.5 wt%.^[^ ^17^, ^18^ ^]^ These nanosheets comprise a 2D hexagonal boron network characterized by three‐center two‐electron B─H─B bonds. HB nanosheets exhibit remarkable multifunctionality: they serve as efficient solid acid catalysts,^[^ ^19^, ^20^ ^]^ strong reducing agents,^[^ ^21^, ^22^, ^23^, ^24^, ^25^, ^26^ ^]^ and even materials capable of deactivating pathogens, including the Omicron variant of SARS‐CoV‐2, influenza virus, feline calicivirus, and bacteriophages.^[^ ^27^ ^]^ Unlike many boron‐based hydrides, HB nanosheets are chemically stable in aqueous environments^[^ ^28^, ^29^ ^]^ and remain solid at room temperature, allowing safe and practical handling.

A key feature of HB nanosheets is their ability to release molecular hydrogen (H_2_) in response to external stimuli. Thermally induced hydrogen release begins at ≈393 K and continues over a wide temperature range up to 1473 K.^[^ ^17^, ^18^ ^]^ Photoinduced release has been demonstrated under UV irradiation^[^ ^30^ ^]^ and, after molecular modifications, under visible light exposure.^[^ ^31^, ^32^, ^33^ ^]^ Hydrogen release can also be triggered electrochemically at low potentials.^[^ ^34^ ^]^ While these stimuli‐responsive behaviors enable controllable hydrogen release, ensuring stability under thermal and light exposure is critical to prevent unintentional hydrogen evolution—particularly in environments where such stimuli are unavoidable, such as outdoor or high‐temperature settings. For instance, during the transport of HB‐based hydrogen carriers under sunlight or elevated temperatures, it is essential to maintain both structural and chemical stability. Likewise, in neutron‐shielding applications—where the excellent neutron attenuation properties of boron and hydrogen are advantageous—preserving material integrity is critical to achieving consistent performance.

Various approaches have been explored to regulate hydrogen release behavior.^[^ ^30^, ^31^, ^32^, ^33^, ^34^, ^35^ ^]^ For instance, tuning the BHB/BH ratio within the HB lattice has been shown to control UV‐induced hydrogen release.^[^ ^35^ ^]^ Isotopic labelling studies have further revealed that thermally activated hydrogen release proceeds primarily via interlayer hydrogen recombination, which is highly sensitive to interlayer spacing.^[^ ^36^ ^]^ Because hydrogen release requires H─H bond formation, increasing the interlayer distance can suppress recombination and potentially enhance both thermal and photostability. However, molecular intercalation strategies must be designed carefully, as certain intercalants can act as photosensitizers and promote unintended light‐driven hydrogen release.^[^ ^31^, ^32^ ^]^ Recently, Ito et al. used graphene to expand the HB interlayer spacing and achieved partial control over the hydrogen‐release temperature, but the effect was limited, and photostability was not examined.^[^ ^36^ ^]^ These observations highlight the need for an intercalant that can effectively expand the HB interlayer spacing while avoiding photosensitization, thereby simultaneously improving thermal and photostability.

In this study, we introduce pyrazine, a nitrogen‐containing heteroaromatic compound, as a non‐photoactive intercalant to improve both the thermal stability and photostability of HB nanosheets. Pyrazine was selected for its wide HOMO–LUMO gap and low propensity for photoexcitation under visible light, minimizing the risk of light‐induced hydrogen release. We synthesized pyrazine‐intercalated HB nanosheets via a simple solution‐phase process and found that even at a low pyrazine content (≈2.9 mol%), the material exhibited a significant improvement in thermal stability, with the hydrogen‐release main peak temperature increasing by ≈200 K, reaching 733 K compared to pristine HB (533 K). These results demonstrate a promising molecular interface engineering strategy for enhancing the environmental robustness of HB‐based hydrogen storage materials.

## 2 Results and Discussion

### 2.1 Synthesis of Pyrazine‐HB

**Figure 1:**
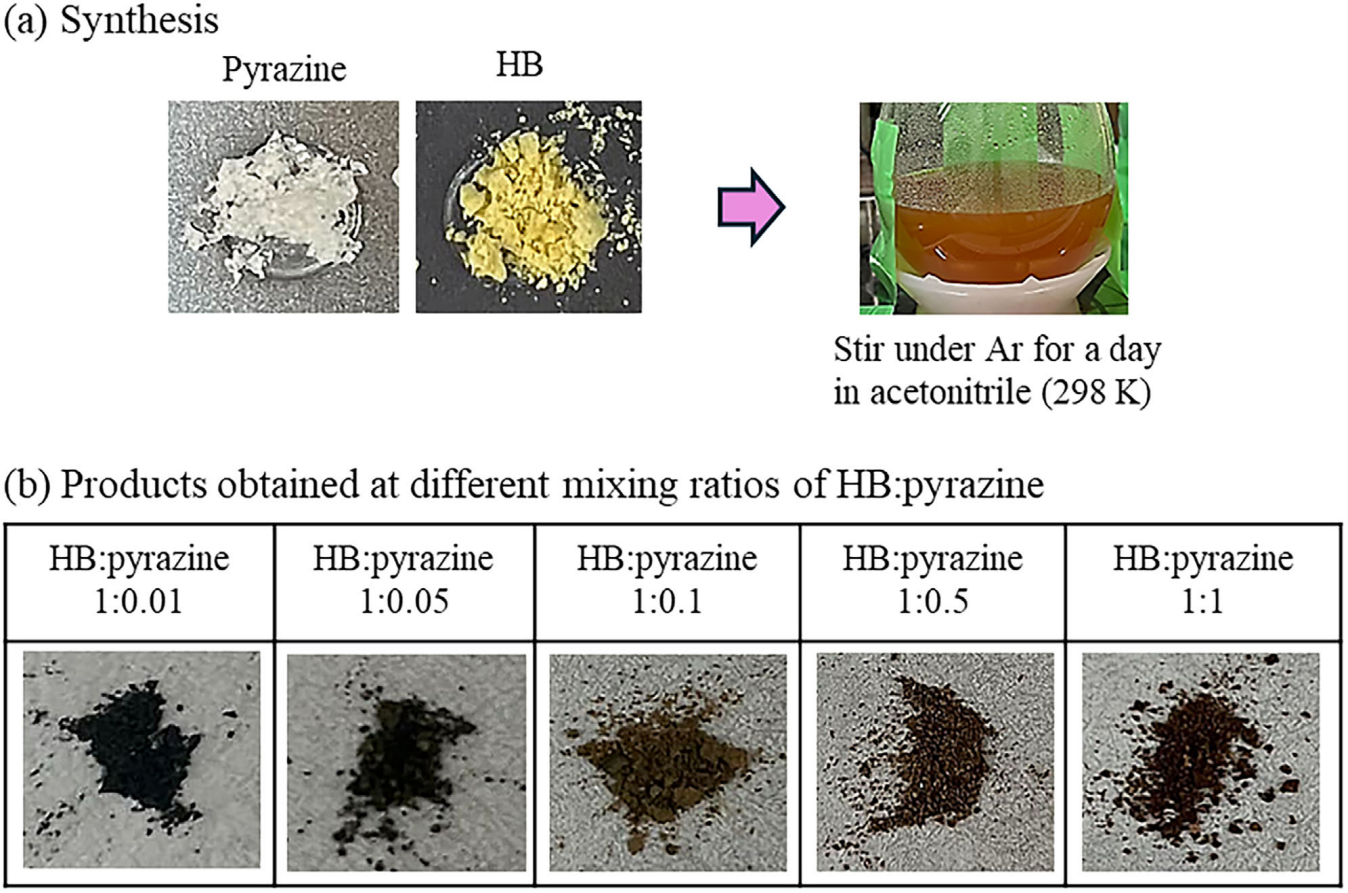
Synthesis of pyrazine‐incorporated hydrogen boride (Pyrazine‐HB) nanosheets. a) Photographs of pyrazine and HB powders and the synthesis procedure involving solution‐phase mixing of pyrazine and HB nanosheets in acetonitrile. b) Photographs of the Pyrazine‐HB powders prepared at different pyrazine contents.

The Pyrazine‐HB nanosheets were synthesized by mixing pristine HB nanosheets with pyrazine at various molar ratios (HB:pyrazine = 1:0.01, 1:0.05, 1:0.1, 1:0.5, and 1:1). In a typical preparation, HB nanosheets dispersed in acetonitrile were combined with a pyrazine solution in acetonitrile and stirred for 24 h under an Ar atmosphere at 298 K (**Figure** **1**a). After acetonitrile removal under reduced pressure and subsequent vacuum drying at 343 K, the obtained powders (Figure 1b) were handled and stored in an Ar‐filled glove box to avoid exposure to air and moisture.

### 2.2 Characterization of Pyrazine‐HB

#### 2.2.1 Thermogravimetric (TG) and Differential Thermal Analysis (DTA)

**Figure 2:**
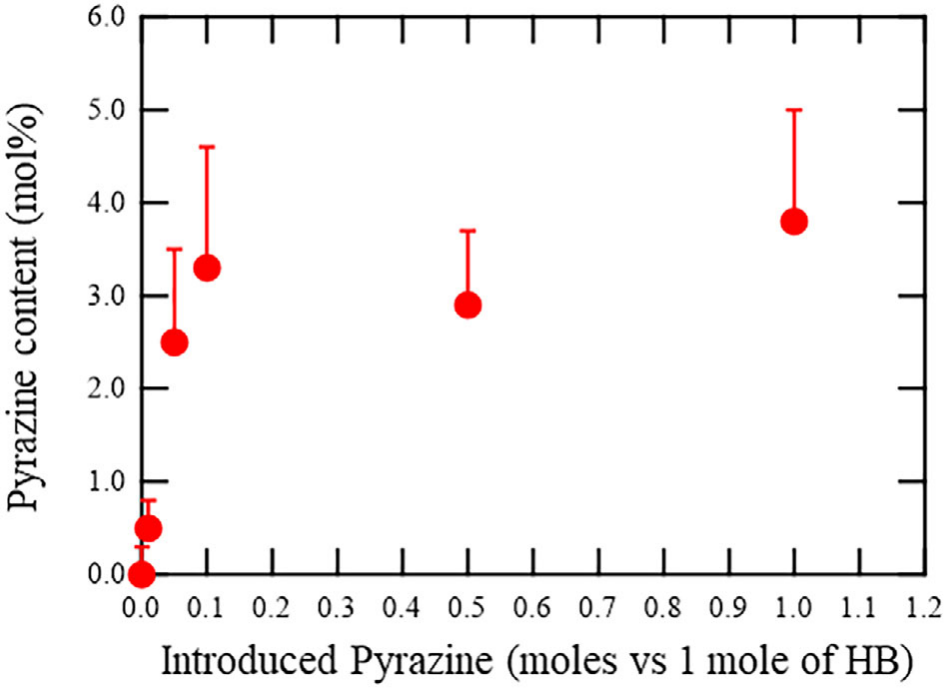
Pyrazine contents of Pyrazine‐HB samples prepared at different Pyrazine:HB ratios.

The red curves in Figure S1a–f (Supporting Information) show the experimental TG profiles of the Pyrazine‐HB samples. To isolate the mass loss attributable to hydrogen desorption, the corresponding hydrogen evolution data (H_2_‐temperature‐programmed desorption, H_2_‐TPD) were used to calculate the theoretical hydrogen‐only weight loss, plotted as blue curves in Figure S1a–f (Supporting Information). In all cases, the overall mass loss exceeded the contribution from hydrogen release alone, confirming the presence of additional volatile species such as H_2_O. In pristine HB, the minor weight loss near 373 K is associated with desorption of physisorbed water, while hydrogen release begins above 473 K. In contrast, the Pyrazine‐HB samples exhibit more pronounced weight loss starting at ≈673 K, and the magnitude increases with higher initial pyrazine content. This behavior suggests that the incorporated pyrazine molecules are thermally released at temperatures above ≈673 K, which is more than 300 K higher than the evaporation temperature of pure pyrazine powder (Figure S1g, Supporting Information). Importantly, after heating to 1473 K in the TG measurements, m/z = 28 mass signals were detected in all samples except pristine HB and HB:pyrazine = 1:0.01 (Figure S2a–f, Supporting Information). This indicates that residual pyrazine molecules decomposed, releasing N_2_ (m/z = 28) as a decomposition product. The actual pyrazine content of each sample was then estimated by subtracting the minimum value of the blue curve (hydrogen‐derived mass loss) from the minimum value of the red curve (total mass loss), as shown in Figure S3 (Supporting Information). The background increase was used as the error bar range in the summarized results in **Figure** **2**. It should be noted that this estimation does not account for water potentially present in the sample or for possible residues of pyrazine decomposition products other than N_2_. The former may lead to an overestimation, while the latter may result in an underestimation of the pyrazine content. Based on these results, the following nomenclature is used to represent the samples: Pyrazine (0.5 mol%)‐HB for HB:Pyrazine = 1:0.01, Pyrazine (2.5 mol%)‐HB for 1:0.05, Pyrazine (3.3 mol%)‐HB for 1:0.1, Pyrazine (2.9 mol%)‐HB for 1:0.5, and Pyrazine (3.8 mol%)‐HB for 1:1 (Figure 2).

The DTA curves of the Pyrazine‐HB samples are shown in Figure S4 (Supporting Information). In the case of pristine HB, two exothermic peaks related to hydrogen release are observed, which is consistent with the TG results. For pure pyrazine sample, endothermic peaks corresponding to melting and volatilization are observed at 329 and 353 K, respectively (Figure S4g, Supporting Information). The peaks of Pyrazine‐HB depend on the pyrazine content. When the ratio of HB to pyrazine is ≥1:0.05 (Pyrazine (2.5 mol%)‐HB), an exothermic peak appears at ≈446 K, and no endothermic peaks are observed. These results suggest that Pyrazine‐HB is not a physical mixture of the two components but a different compound formed by the interaction between pyrazine molecules and HB.

#### 2.2.2 Fourier Transform‐Infrared Absorption Spectroscopy (FT‐IR) and X‐Ray Photoelectron Spectroscopy (XPS)

**Figure 3:**
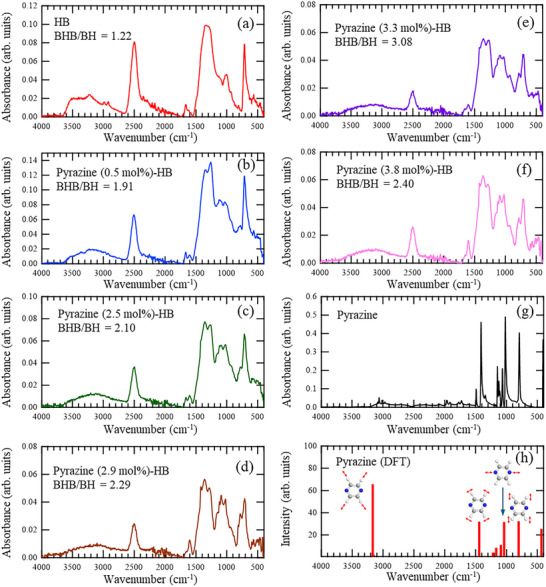
Fourier transform‐infrared spectra of a) Pristine HB, b) Pyrazine (0.5 mol%)‐HB, c) Pyrazine (2.5 mol%)‐HB, d) Pyrazine (2.9 mol%)‐HB, e) Pyrazine (3.3 mol%)‐HB, f) Pyrazine (3.8 mol%)‐HB, and g) pure pyrazine. h) FT‐IR spectrum of pyrazine calculated by density functional theory.

The FT‐IR spectra of the Pyrazine‐HB samples are shown in **Figure** **3**a–g. The characteristic absorption band near 2500 cm^−1^ is attributed to the B–H stretching vibrational mode of terminal BH units, while the peak at ≈1360 cm^−1^ originates from the B–H–B bridge structures.^[^ ^35^, ^36^, ^37^ ^]^ As the pyrazine content increases, the relative intensity of the BHB‐associated band at 1360 cm^−1^ becomes higher than that of the B–H peak. This apparent enhancement is likely due to spectral overlap with the pyrazine‐derived peak (Figure 3g). Density functional theory (DFT) calculations (Figure 3h) indicate that the bands at 1412 and 792 cm^−1^ correspond to the C─H vibrations on the pyrazine ring. Importantly, the persistence of these C─H bands in the experimental spectra suggests that the hydrogen atoms on the pyrazine ring remain chemically intact during synthesis. These observations imply that pyrazine interacts with HB via its nitrogen atoms rather than its hydrogen atoms.

To further examine the interaction between pyrazine and HB, X‐ray photoelectron spectroscopy (XPS) measurements were conducted for pristine HB and Pyrazine‐HB (3.3 mol%), as shown in Figure S5 (Supporting Information). For pristine HB, the main B 1s peak appears at 187 eV, consistent with negatively charged boron as previously reported.^[^ ^17^, ^18^ ^]^ A small peak at 191.5 eV is attributable to the oxidized species in the HB surface and/or by‐product boric acid.^[^ ^17^ ^]^ In Pyrazine–HB, a distinct N 1s peak is observed at 400.5 eV, indicating that nitrogen in the pyrazine molecule is slightly positively charged compared to pyridinic nitrogen (398.5 eV).^[^ ^38^ ^]^ This result suggests that pyrazine interacts with HB via its nitrogen atoms, possibly involving hydrogen in the BHB units. Similar interaction was previously reported between HB and nitrogen‐containing organic heterocycles,^[^ ^32^ ^]^ where the density functional theory in that report shows the presence of protonated nitrogen‐containing heterocycles, formed by the donation of protons by HB. Consistently, in Pyrazine–HB, the main B 1s peak and the minor oxide peak shift to 188.0 and 192.5 eV, respectively, i.e., ≈1.0 eV higher than those of pristine HB (Figure S6, Supporting Information). These shifts are likely caused by charge transfer from pyrazine to HB during protonation of pyrazine, which raises the Fermi level and results in a higher binding energy for B 1s.

#### 2.2.3 X‐Ray Diffraction (XRD)

The XRD patterns of the Pyrazine‐HB samples are shown in Figure S6a–f (Supporting Information). No distinct crystalline peaks are observed in any of the samples, indicating that the introduction of pyrazine does not induce crystallinity within the HB nanosheet structure. In contrast, the XRD pattern of pure pyrazine (Figure S6g, Supporting Information) displays sharp diffraction peaks, confirming its high crystallinity. The absence of sharp peaks in the Pyrazine‐HB samples suggests that pyrazine is homogeneously dispersed or intercalated within the disordered HB nanosheets and does not form separate crystalline domains.

#### 2.2.4 UV–Vis Spectra and Optical Appearance

**Figure 4:**
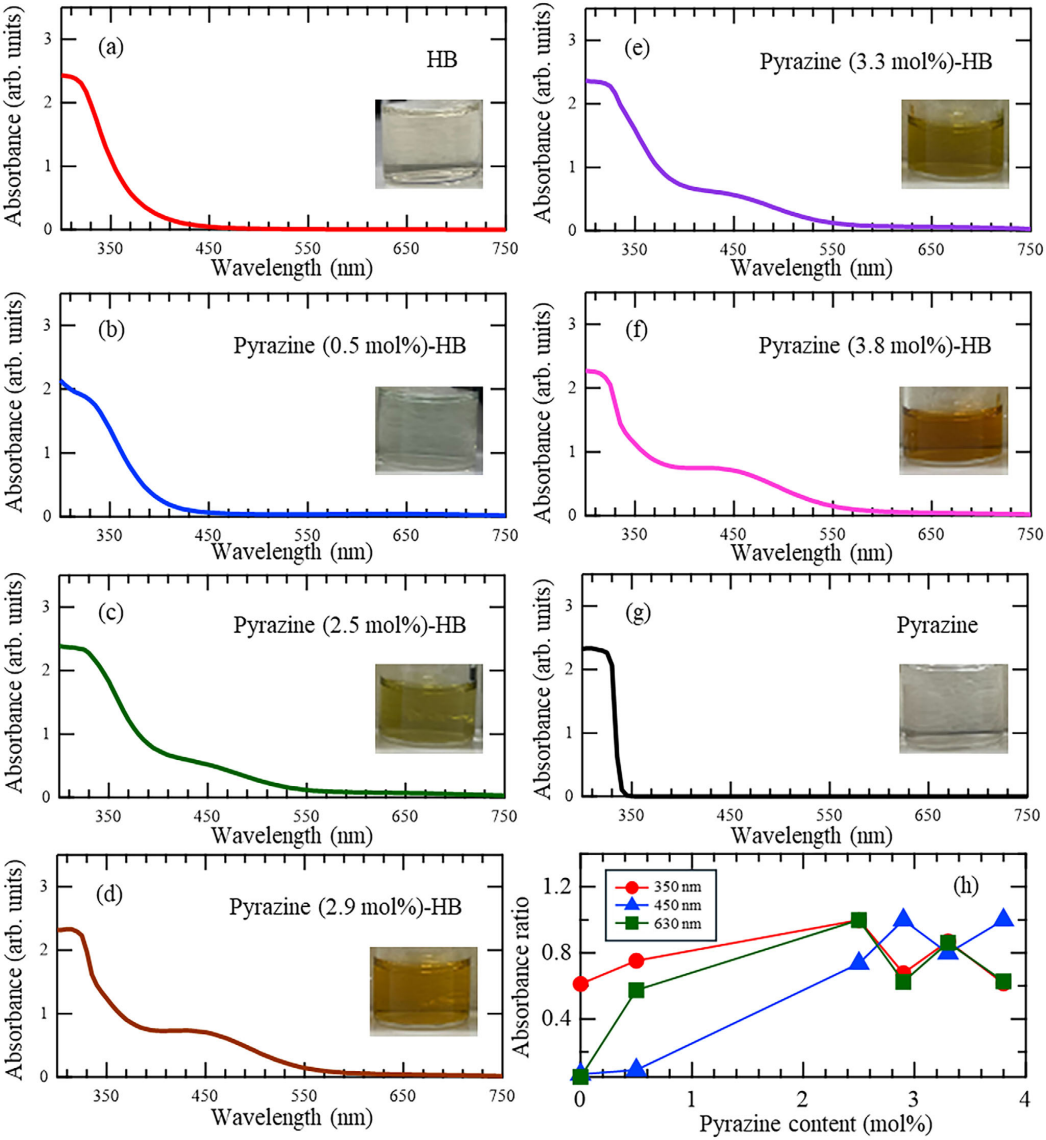
Optical appearance and UV–vis absorption spectra of a) pristine HB, b) Pyrazine (0.5 mol%)‐HB, c) Pyrazine (2.5 mol%)‐HB, d) Pyrazine (2.9 mol%)‐HB, e) Pyrazine (3.3 mol%)‐HB, f) Pyrazine (3.8 mol%)‐HB, and g) pure pyrazine. h) Normalized absorbance values of all the samples at 350, 450, and 630 nm (normalized to a maximum value of 1 at each wavelength).

The UV–vis absorption spectra and visual color appearances of the Pyrazine‐HB samples are shown in **Figure** **4**a–g. All the measurements were performed using 1 mg mL^−1^ dispersion liquids in acetonitrile. Figure 4h shows the normalized absorbance intensities at 350, 450, and 630 nm, each scaled to a maximum value of 1 for direct comparison of the samples. The color of Pyrazine (0.5 mol%)‐HB dispersion liquid gradually changes from bluish to green and then to orange with increasing pyrazine content. These color changes correlate with systematic variations in the UV–vis absorption spectra. In the low concentration range (0.5–2.5 mol% pyrazine), the absorbance peaks at 350 and 630 nm intensify with increasing pyrazine content. However, at higher pyrazine loadings (≥2.5 mol%), the intensities at 350 and 630 nm begin to slightly decrease, and a new band appears at 450 nm. Notably, neither HB nor pure pyrazine exhibits absorption at 450 or 630 nm. The appearance of this new band in the Pyrazine‐HB samples suggests that Pyrazine and HB are not merely a physical mixture but interact electronically with each other. These spectral features imply a change in the electronic structure of HB upon molecular interaction or intercalation with pyrazine, which is consistent with XPS results (Figure S6, Supporting Information).

#### 2.2.5 Photoluminescence (PL) Behavior

**Figure 5:**
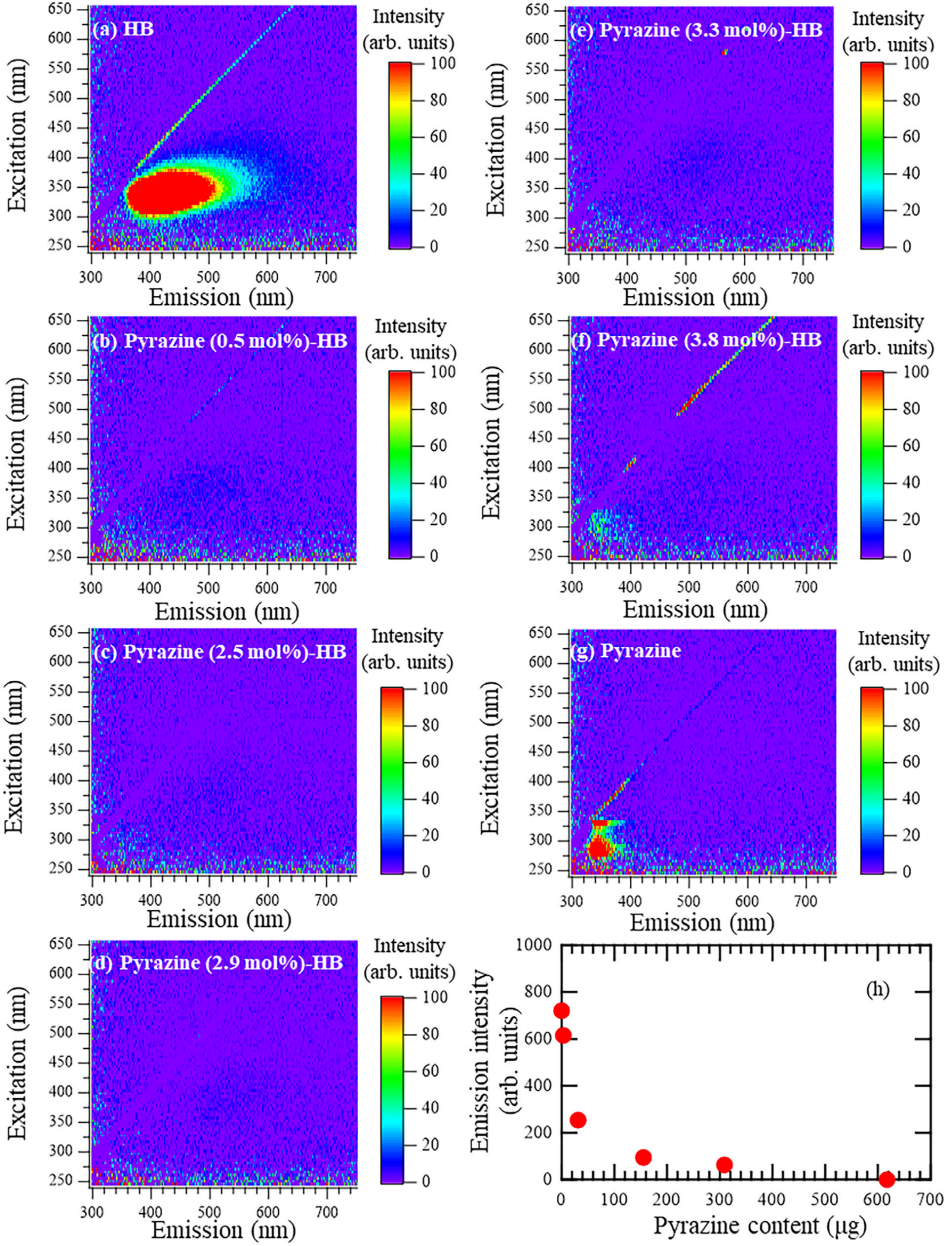
Excitation emission matrix fluorescence analysis results of a) pure HB, b) Pyrazine (0.5 mol%)‐HB, c) Pyrazine (2.5 mol%)‐HB, d) Pyrazine (2.9 mol%)‐HB, e) Pyrazine (3.3 mol%)‐HB, f) Pyrazine (3.8 mol%)‐HB, and g) pure pyrazine. h) Variation of fluorescence intensity under 340 nm excitation light with the addition of pyrazine to a 1 mg mL^−1^ dispersion liquid of HB in acetonitrile, and allowing it to stand for 10 min.

The PL properties of the Pyrazine‐HB samples were evaluated using excitation–emission matrix (EEM) spectroscopy, and the results are summarized in **Figure** **5**. Pristine HB and pure pyrazine exhibit emission peaks at 420 and 345 nm, respectively, under UV excitation (Figure 5a,g). Notably, even a minimal pyrazine content (0.5 mol%) causes complete quenching of the HB emission, and this quenching effect persists up to 3.3 mol% (Figure 5b–e). On the other hand, a faint emission peak resembling that of pure pyrazine is observed in the spectrum of Pyrazine‐HB with 3.8 mol% pyrazine (Figure 5f), which may originate from a small amount of dissolved pyrazine in acetonitrile from Pyrazine‐HB. The complete suppression of HB luminescence by trace amounts of pyrazine prompted further investigation into its potential sensing capability. Upon adding 3 µg of pyrazine to a 1 mg mL^−1^ HB dispersion liquid in acetonitrile (corresponding to 0.04 mol% pyrazine for HB) and followed by a 10‐min equilibration period, the emission intensity decreased (Figure 5h). This result highlights the high sensitivity of HB to pyrazine and the feasibility of employing HB nanosheets as fluorescent sensors for nitrogen‐containing heterocycles. Further studies are warranted to determine the sensing limits, selectivity, and dynamic responses.

### 2.3 Thermal Stability of Pyrazine‐HB

#### 2.3.1 Hydrogen Temperature‐Programmed Desorption (H_2_‐TPD) Analysis

**Figure 6:**
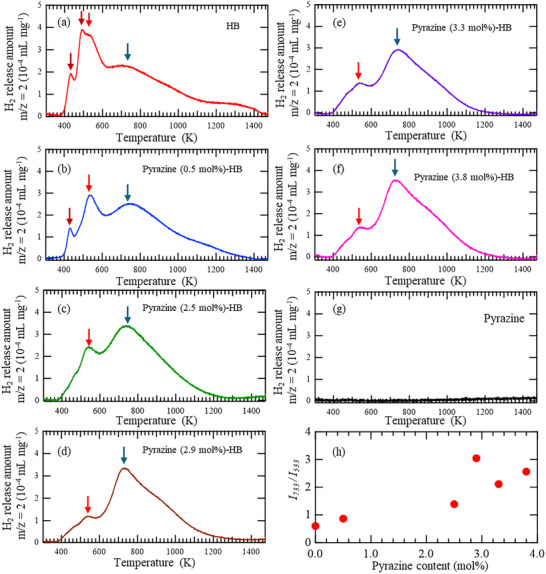
Hydrogen temperature‐programmed desorption (H_2_‐TPD) profiles of a) pristine HB, b) Pyrazine (0.5 mol%)‐HB, c) Pyrazine (2.5 mol%)‐HB, d) Pyrazine (2.9 mol%)‐HB, e) Pyrazine (3.3 mol%)‐HB, f) Pyrazine (3.8 mol%)‐HB, and g) pure pyrazine. The TPD signals were normalized to mL mg^−1^ based on calibration with standard hydrogen gas. h) Relationship between the pyrazine content and intensity ratio of TPD peaks at 533 and 733 K. In panels a–f, red and blue arrows represent the 533 and 733 K peaks, while brown arrows in a and b represent peak components due to different stacking distances of HB nanosheets.^[^ ^36^ ^]^

The H_2_‐TPD profiles of pristine HB, Pyrazine‐HB samples, and pure pyrazine are shown in **Figure** **6**a–g. In the pristine HB sample, prominent desorption peaks appear at ≈423 and 533 K. With increasing pyrazine content, these low‐temperature desorption peaks are markedly suppressed, while a new high‐temperature desorption peak emerges and intensifies near 733 K. The ratio of the desorption intensity at 733 K to that at lower temperatures increases by approximately threefold compared with that of pristine HB (Figure  6h). This substantial modulation of the desorption profile was achieved even at a low pyrazine content of ≈3 mol%. Such a drastic change in hydrogen release behavior cannot be adequately explained by a simple physical barrier effect, such as surface capping by pyrazine. Instead, the introduction of pyrazine induces electronic and/or structural perturbations within the HB nanosheet framework, which inhibit hydrogen recombination and alter the hydrogen‐binding environment. These results indicate a fundamental shift in the desorption mechanism induced by molecular‐level modification, underscoring the critical role of intercalated species in tuning hydrogen storage performance. The detailed origin of the change in the TPD profile, such as the disappearance of the 423 K peak with increasing pyrazine content, is discussed below.

#### 2.3.2 Origin of Thermal Stability

Ito et al. reported that hydrogen release from HB nanosheets under thermal conditions primarily arises from the recombination of hydrogen atoms located between the stacked HB layers, rather than within individual sheets.^[^ ^36^ ^]^ Therefore, the interlayer distance between the nanosheets is a key parameter governing the hydrogen release temperature. Increasing the interlayer spacing inhibited the stacking of HB nanosheets, decreasing the probability of hydrogen recombination, thereby suppressing desorption of H_2_ at lower temperatures. In this study, the significant increase in the hydrogen release temperature observed with the incorporation of trace amounts of pyrazine suggests that the pyrazine molecules act as effective interlayer spacers, preventing close stacking of the HB nanosheets. Similar intercalation effects of pyrazine molecules in layered material (layered copper nitroprusside) have also been reported previously.^[^ ^39^ ^]^ As a result of pyrazine intercalation in stacked HB nanosheets, the low‐temperature H_2_‐release peaks in TPD (423 and 533 K, which are attributed to hydrogen release from layers with different stacking distances)^[^ ^36^ ^]^ are considered to progressively disappear with increasing pyrazine content (Figure 6).

To validate this hypothesis, we measured the Brunauer–Emmett–Teller (BET) surface areas of the pristine HB and Pyrazine‐HB samples. As shown in Figure S7 (Supporting Information), the surface area of pristine HB is negligibly small, likely owing to aggregation during the drying process. In contrast, Pyrazine‐HB exhibited a BET surface area of 17 m^2^ g^−1^, indicating enhanced accessibility of the nanosheet surfaces. This expansion of the accessible surface area strongly supports the interpretation that the pyrazine molecules increase the interlayer distance between the HB nanosheets. This structural modification reduces the interlayer hydrogen recombination pathways and enhances the thermal stability of Pyrazine‐HB.

To directly confirm intercalation, transmission electron microscopy (TEM) and scanning transmission electron microscopy (STEM) observations were conducted, along with elemental mapping by energy‐dispersive X‐ray spectroscopy (EDS), for both pristine HB and Pyrazine‐HB samples (Figures S8 and S9, Supporting Information). For pristine HB, the line profile at folded‐layer sites shows clear lattice space of 3.4 Å, which is consistent with theoretically calculated layer‐layer distance of the “hollow‐stacking HB nanosheets”^[^ ^17^ ^]^ (Figure S8a, Supporting Information), whereas in Pyrazine–HB, a range of interlayer distances from 3.4 to 5.0 Å is observed (Figure S8b–d, Supporting Information). This indicates that pyrazine intercalation introduces fluctuations in the layer‐to‐layer spacing. Consistently, elemental mapping shows highly dispersed nitrogen throughout the Pyrazine‐HB sample, whereas an elemental mapping of the pristine‐HB sample does not show (Figure S9, Supporting Information).

These findings collectively demonstrate that pyrazine incorporation modulates the stacking structure of HB nanosheets by expanding and inducing fluctuations in the interlayer spacing. This structural modification reduces interlayer hydrogen recombination pathways, thereby enhancing thermal stability and shifting hydrogen release to higher temperatures.

**Figure 7:**
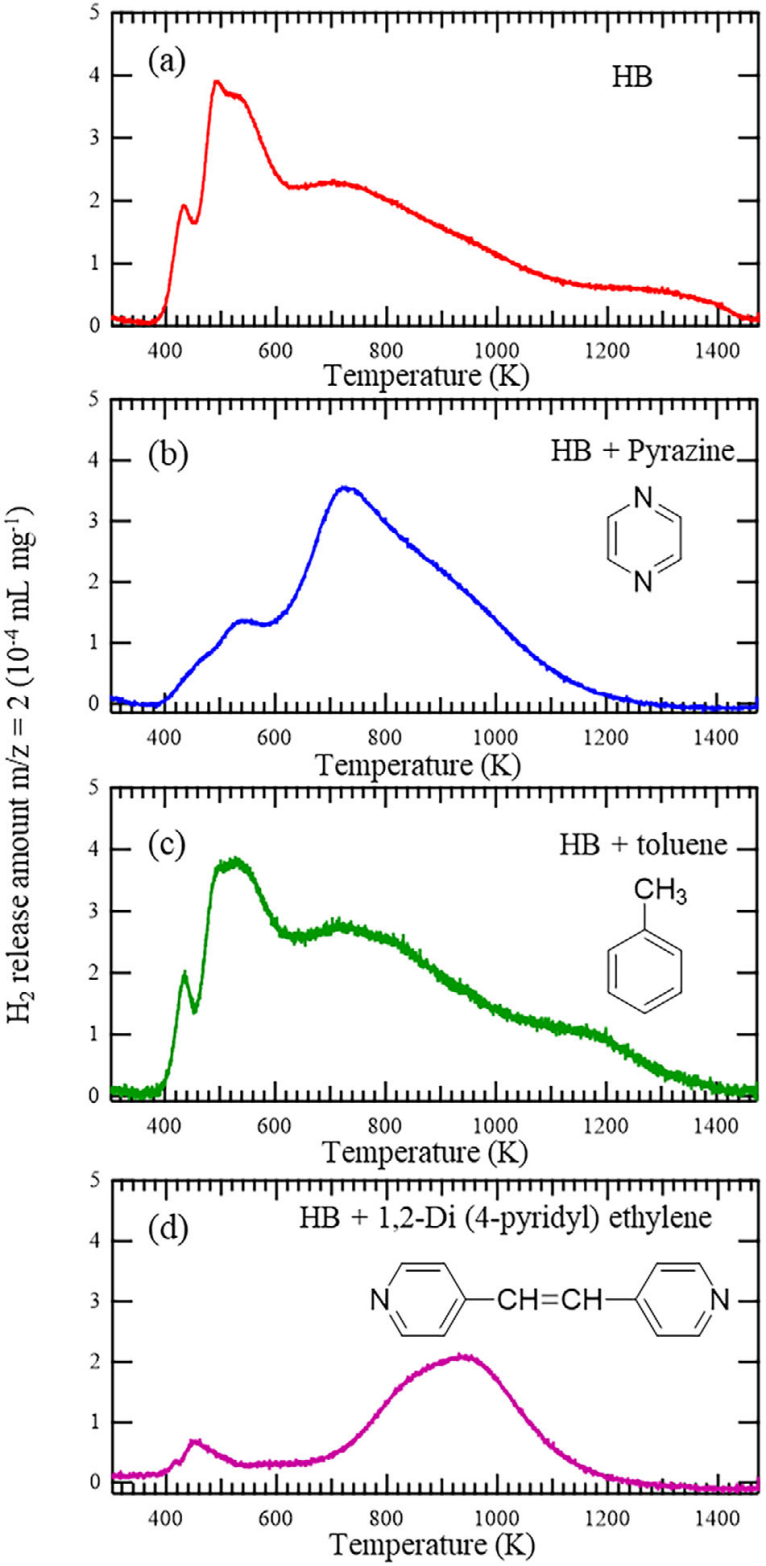
Thermogravimetric and temperature‐programmed desorption analysis of a) pristine HB, b) Pyrazine‐HB, c) toluene‐HB, and d) 1,2‐di(4‐pyridyl)ethylene‐HB.

To examine the nature of the interactions responsible for the enhanced thermal stability and altered stacking behavior of HB nanosheets with pyrazine incorporation, we conducted comparative experiments using two control molecules: toluene, a non‐nitrogen‐containing aromatic compound with a molecular size similar to that of pyrazine, and 1,2‐di(4‐pyridyl)ethylene (DPE), a bulkier nitrogen‐containing heterocycle. The corresponding TPD profiles are presented in **Figure** **7**. The hydrogen release temperature of HB significantly increases with the incorporation of nitrogen‐containing heterocycles such as pyrazine and DPE. In contrast, the introduction of toluene does not significantly change the thermal behavior of HB. These results suggest that nitrogen plays an important role in mediating the interaction between HB and the additive as an intercalant. However, because of the limitations of the present experimental design, we could not fully separate the effects of molecular size and polarity. A more systematic study using a broader range of control molecules with varied sizes and functional groups will be necessary to clarify the factors that contribute most strongly to effective intercalation behavior. As supported by the FT‐IR and XPS analysis discussed earlier, this interaction is likely governed by the hydrogen bonding between the nitrogen atoms of the heterocycle and acidic protons of B–H–B on the HB surface. Notably, the increase in the hydrogen release temperature in DPE‐HB is more pronounced than that of Pyrazine‐HB. This may be attributed to the larger steric volume of DPE, which induces greater interlayer separation within the HB structure. Alternatively, DPE may influence the electronic environment of the HB framework through π–π or donor–acceptor interactions, contributing to the observed stabilization. These mechanistic pathways are currently under investigation and will be explored in future studies.

### 2.4 Photostability of Pyrazine‐HB

**Figure 8:**
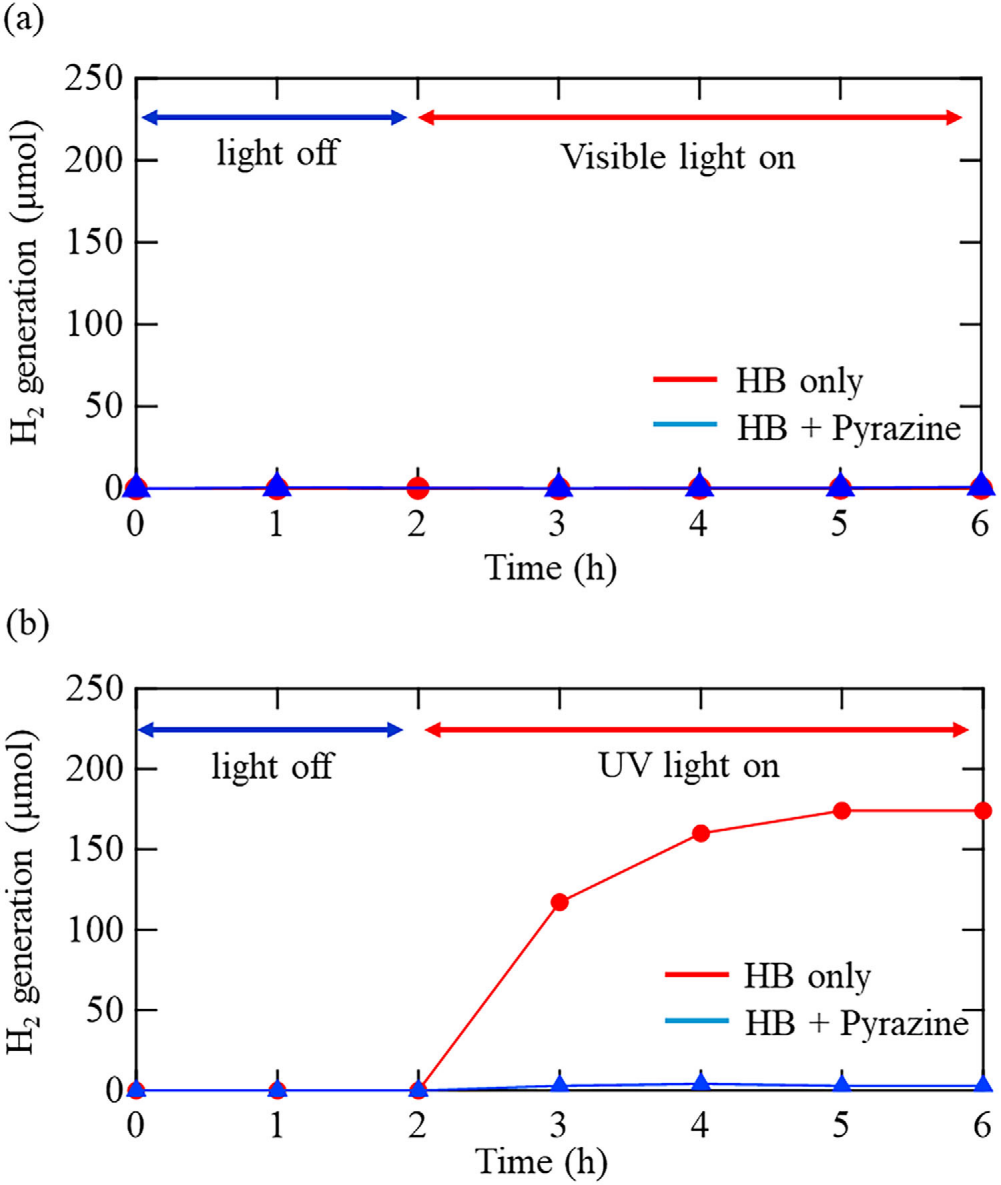
Hydrogen evolution profiles of HB and Pyrazine‐HB in acetonitrile (pyrazine: 10 µmol, HB: 24 µmol, total volume: 5 mL) under a) visible light and b) UV light irradiation.

The photoinduced hydrogen evolution behavior of pristine HB and Pyrazine‐HB was investigated under visible‐light irradiation, as shown in **Figure** **8**a. Neither sample exhibited measurable hydrogen evolution under visible light. The large HOMO–LUMO gap of pyrazine (≈5.8 eV)^[^ ^40^ ^]^ prevents excitation by the visible light source used in this study (Figure S10, Supporting Information). As shown in Figure 4, Pyrazine‐HB samples absorbed visible light in the 450–630 nm range, which overlaps with the emission spectrum of the light source. Nevertheless, no hydrogen release was observed under visible‐light irradiation, as shown in Figure 8a. Takeshita et al. previously reported that visible‐light‐induced hydrogen release from nitrogen‐containing heterocycles like phenanthroline^[^ ^32^ ^]^ occurs when HB donates protons and electrons to form radical species that facilitate hydrogen desorption via additional proton reduction. They also showed that the hydrogen release rate under visible light irradiation strongly depends on the proton and electron affinities of the heterocycles. These findings suggest that, in the present Pyrazine‐HB system, proton transfer is taking place as indicated by FT‐IR and XPS, but electron transfer or additional proton reduction is probably suppressed, preventing hydrogen evolution.

Under UV light, pristine HB shows clear H_2_ release, consistent with previous reports.^[^ ^30^ ^]^ In contrast, Pyrazine‐HB exhibits a strongly suppressed hydrogen evolution rate, demonstrating that pyrazine incorporation enhances HB photostability (Figure 8b). According to a previous report,^[^^30^^]^ hydrogen release from HB under UV irradiation is driven by band‐to‐band excitation and electron injection into the antibonding state of hydrogen in HB. As shown in the fluorescence spectra (Figure 5), the excitation and recombination processes of HB are strongly influenced by pyrazine incorporation, indicating that UV‐induced hydrogen release is highly dependent on the modified electronic environment introduced by pyrazine.

Analogous to the mechanism proposed for enhanced thermal stability, the suppression of hydrogen release under UV irradiation in Pyrazine‐HB can be partially attributed to the increased interlayer spacing, which hinders the recombination of hydrogen atoms located between nanosheets. It is worth noting that HB nanosheets dispersed in acetonitrile form aggregates, and their size depends on the HB concentration in acetonitrile, as reported by dynamic light scattering measurements.^[^ ^35^ ^]^ Thus, pyrazine intercalation can modulate the photoinduced interlayer hydrogen recombination pathway even in acetonitrile. According to a previous report, pristine HB can release nearly its entire hydrogen content (up to 8.0 wt%) under UV irradiation at room temperature,^[^ ^30^ ^]^ suggesting that hydrogen recombination can also occur within individual HB layers under photoexcitation. UV‐excited HB nanosheets dissipate energy through two primary channels: photoluminescence and hydrogen release, and the relative contributions of these pathways can be tuned by modifying the structural motif, specifically, the ratio of bridging BHB to terminal BH units.^[^ ^35^ ^]^ As shown in Figure 4, pyrazine incorporation enhances light absorption, yet photoluminescence is completely quenched in all Pyrazine‐HB samples (Figure 5), even at the lowest incorporation levels. This indicates that the optical properties of HB are drastically altered by trace amounts of pyrazine. If photoluminescence and hydrogen release were the only available energy‐dissipation pathways, suppression of photoluminescence would be expected to enhance hydrogen release. However, hydrogen release is also suppressed and shifted to higher temperatures (Figure 6), implying the activation of a third, previously inactive energy‐dissipation pathway upon pyrazine incorporation. Possible mechanisms include nonradiative relaxation via the vibrational modes of pyrazine or transient interactions such as N─H bond formation, which warrant further investigation through in situ spectroscopic studies under UV excitation. The absence of hydrogen evolution under visible light and the suppressed release under UV illumination demonstrate the dual optical stability of Pyrazine–HB, underscoring the role of pyrazine as a non‐photoactive stabilizing intercalant.

## 3 Conclusion

In summary, we developed thermally stable and photostable Pyrazine‐HB nanosheets via a facile solution‐based approach by mixing HB nanosheets with trace amounts of pyrazine in acetonitrile. The resulting material retained a high hydrogen content while exhibiting a significant increase in the hydrogen desorption temperature, as confirmed by TG‐DTA‐TPD analysis. Moreover, Pyrazine‐HB exhibited strong resistance to UV irradiation, suppressing both hydrogen release and photoluminescence. These enhancements are attributed to the structural and electronic effects of pyrazine incorporation, including increased interlayer spacing and the formation of alternative energy‐dissipation pathways. Thus, molecular intercalation is an effective strategy for stabilizing HB nanosheets and expanding their practical application in environments that require thermal and photostability. Furthermore, we demonstrated the feasibility of employing HB nanosheets as highly sensitive fluorescent sensors for nitrogen‐containing heterocycles, detecting pyrazine concentrations as little as 0.04 mol% via fluorescence changes. This study provides a foundation for designing functionalized HB‐based materials for hydrogen storage, high‐sensitivity sensors, and neutron shielding materials. Future studies will investigate the sensing limits, selectivity, and dynamic responses of Pyrazine‐HB, as well as explore its energy‐dissipation mechanisms using in situ spectroscopic analysis under UV irradiation.

## 4 Experimental Section

### Synthesis of HB Nanosheets

Hydrogen boride (HB) nanosheets were synthesized using a previously reported ion‐exchange method.^[^ ^17^, ^18^, ^37^ ^]^ Magnesium diboride (MgB_2_, Pavtec Co., Ltd.) was used as the precursor, and proton exchange was carried out using a strongly acidic cation exchange resin (Amberlite IR120B, H⁺ form; Organo Corporation). In a typical procedure, MgB_2_ powder (1.0 g) was added to acetonitrile (200 mL; special grade; FUJIFILM Wako Pure Chemical Corporation) containing the ion exchange resin (60 mL) in a Schlenk flask. The mixture was magnetically stirred at 310 rpm in Ar (≈1 atm) at ≈300 K for 72 h. After completion of the exchange reaction, the resulting mixture was vacuum‐filtered through a 0.2‐µm membrane to remove the black precipitate. The filtrate was then cooled to 255 K and left to stand for 12 h to precipitate boric acid, a by‐product. A second filtration was performed at room temperature (≈300 K), and the filtrate was subsequently concentrated under reduced pressure at 313 K in an oil bath under an Ar atmosphere. The concentrated dispersion liquid was dried at 343 K for 30 min to obtain a yellow HB nanosheet powder. Acid‐assisted exfoliation methods^[^ ^41^ ^]^ were avoided to prevent contamination by residual acid species. Additionally, pretreatment protocols for long‐term stability enhancement against water^[^ ^29^ ^]^ were not applied in this study to ensure a pristine comparison of the structural and functional properties.

### Synthesis of Pyrazine‐HB

Pyrazine‐intercalated HB nanosheets (Pyrazine‐HB) were prepared by mixing HB nanosheets with pyrazine (≥ 97.0%, FUJIFILM Wako Pure Chemical Corporation) at predetermined molar ratios of HB:pyrazine = 1:0.01, 1:0.05, 1:0.1, 1:0.5, and 1:1. In a typical procedure, pyrazine was dissolved in acetonitrile (200 mL; special grade; FUJIFILM Wako Pure Chemical Corporation), and a separately prepared dispersion of HB nanosheets in a few milliliters of acetonitrile was added to the solution. The resulting suspension was magnetically stirred (310 rpm) in the dark for 24 h at 298 K and ambient pressure under an Ar atmosphere. After mixing, the dispersion liquid was concentrated under reduced pressure at 313 K and dried under vacuum at 343 K in an oil bath for 30 min. The filtration step was not performed after the synthesis. The resulting powder samples were stored in an Ar‐filled glove box to prevent degradation by air and moisture.

### Synthesis of Toluene‐HB

Toluene‐intercalated HB nanosheets (toluene‐HB) were synthesized by mixing HB nanosheets with toluene (special grade, FUJIFILM Wako Pure Chemical Corporation) at a molar ratio HB:toluene of 1:1. In a typical procedure, toluene (1.8 mL) was dissolved in acetonitrile (200 mL; special grade), and HB nanosheets (200 mg) predispersed in a few milliliters of acetonitrile were added to the solution. The mixture was magnetically stirred in the dark at 310 rpm for 24 h at 298 K and ambient pressure under an Ar atmosphere. After the reaction, the suspension was concentrated under reduced pressure at 313 K and vacuum‐dried at 343 K for 30 min in an oil bath. The filtration step was not performed after the synthesis. The final powder was stored in an Ar‐filled glovebox to prevent degradation by air and moisture.

### FT‐IR Measurements

The FT‐IR spectra were recorded at 298 K using a Bruker Alpha spectrometer (ALPHA II, Bruker, Billerica, MA, USA) equipped with a diamond‐attenuated total reflectance accessory. All the measurements were performed inside an Ar‐filled glovebox to prevent degradation of air‐ or moisture‐sensitive samples.

### DFT Calculations

Geometry optimizations were performed using Gaussian16 revision C.01,^[^ ^42^ ^]^ without any symmetry constraints. Calculations were performed using the restricted B3LYP functional,^[^ ^43^ ^]^ corrected for dispersion as proposed by Grimme (D3 correction with Becke–Johnson damping),^[^ ^44^, ^45^ ^]^ with the 6‐311G+(d,p) basis set.^[^ ^46^ ^]^ A frequency calculation was performed, and thermodynamic corrections were obtained from frequency analysis, confirming that the optimized structures correspond to minima (with no imaginary frequencies) at 298.15 K. The Cartesian coordinates are provided in the Table S1 (Supporting Information).

### XRD Measurements

The XRD patterns were recorded at 298 K using a benchtop diffractometer (MiniFlex, Rigaku, Tokyo, Japan) equipped with a Cu Kα radiation source generated by a line‐focus tube. The samples were sealed in Kapton capsules inside an Ar‐filled glovebox to prevent exposure to air and moisture prior to measurement. The diffraction data were acquired using a D/teX Ultra silicon strip detector (Rigaku) at a scan rate of 2.0° min^−1^ over a 2θ range up to 90°.

### UV–vis and EEM Measurements

The UV–vis absorption spectra and EEM fluorescence spectra were recorded using a spectrofluorometer (Duetta, HORIBA, Kyoto, Japan). Pyrazine‐HB, pristine HB, and pyrazine samples were each dispersed in acetonitrile at a concentration of 1.0 mg mL^−1^, and all the measurements were carried out at 298 K. The UV–vis and EEM data were recorded simultaneously using the same sample dispersion liquids.

### TG‐DTA and TPD Measurements

The TG‐DTA and TPD measurements were conducted using a thermogravimetric analyzer (STA‐2500 Regulus, NETZSCH, Tokyo, Japan) under an Ar flow of 70  mL min^−1^. The samples were heated from 298 to 1473 K at a rate of 10 K min^−1^. The evolved gases were analyzed using a quadrupole mass spectrometer (Microvision 2, MKS, Ardech, France) connected via a capillary line to a custom‐built ultrahigh vacuum chamber integrated with the TG system. The TPD profiles were monitored for m/z values of 2, 16, 18, 28, 32, 40, 44, and 80. A minor signal at m/z = 16 was observed at ≈728 K, which may be attributed to trace pyrazine‐derived fragments. No other significant peaks corresponding to pyrazine or its decomposition products were observed. The quantification of m/z = 18 (H_2_O) was not reliable owing to condensation inside the capillary line.

### Photoinduced Hydrogen Evolution Measurements

The photoinduced hydrogen evolution was investigated using a quartz reaction vessel containing acetonitrile dispersion liquid (5 mL) with HB (24 µmol) and pyrazine (10 µmol). The dispersion liquid was irradiated from a distance of 8 cm using either visible or UV light while stirring at 250 rpm. UV light irradiation was provided by a 150 W mercury‐xenon (Hg‐Xe) lamp, while visible light irradiation was provided by a 500 W xenon (Xe) lamp equipped with a short wavelength cutoff filter (*λ* < 470 nm cutoff) (Figure S10, Supporting Information). The evolved hydrogen was analyzed using a gas chromatograph (GC‐2014, Shimadzu, Japan) equipped with a thermal conductivity detector. The reaction was maintained at room temperature using a recirculating water chiller.

### Brunauer–Emmett–Teller (BET) Surface Area Measurements

The specific surface area was determined by nitrogen adsorption at 77 K using a BELSORP MAX G (MicrotracBEL, Osaka, Japan). Prior to measurement, the samples were degassed under vacuum at 373 K for 2 h. The BET surface area was determined using the linear region of the BET plot, which was calculated from the adsorption isotherm in the relative pressure (*P*/*P*_0_) range of 0.05–0.30, employing the BELSORP Data Analysis Software (BELMaster, Ver. 7.3.2.0).

### Transmission Electron Microscopy (TEM) Observation

The microstructure and morphology of the HB samples were examined by transmission electron microscopy (TEM). Observations were performed using a TEM instrument (HF5000, Hitachi High‐Tech, Japan) operated at 200 kV and equipped with two energy‐dispersive X‐ray spectroscopy (EDS) detectors (EDAX Elite T, Gatan, USA). The HB powder samples were dispersed on a holey carbon‐supported Cu grid.

### X‐Ray Photoelectron Spectroscopy (XPS) Measurements

XPS measurements were conducted using a JEOL JPS‐9010 TR spectrometer equipped with an ultrahigh vacuum chamber and an Mg Kα X‐ray source (1253.6 eV), with a pass energy of 10 eV. Samples were mounted on In on graphite tape, and the Shirley background was subtracted from the spectrum using SpecSurf version 1.8.3.7 (JEOL, Ltd., Japan). Incomplete contact between the graphite tape and the sample holder caused slight charge build‐up, resulting in marginal shifts of the spectra toward higher binding energies. Therefore, we used the In 3d_5/2_ peak of the substrate In (443.9 eV) as a reference to calibrate for this charge build‐up.

## Conflict of Interest

The authors declare no conflict of interest.

## Supporting information



Supporting Information

## Data Availability

Data sets generated during the current study are available from the corresponding author on reasonable request.
