# Supplementary material for: Enhanced Thermal‐ and Photostability of Trace Pyrazine‐Incorporated Hydrogen Boride Nanosheets
Source: Small. 2025 Oct 22;21(49):e06230. doi: 10.1002/smll.202506230 (PMC12696797; doi:10.1002/smll.202506230)
Supplement: Supplementary file 1 — Supporting Information [file SMLL-21-e06230-s001.docx]

Supporting Information

**Enhanced Thermal- and Photostability of Pyrazine-Incorporated Hydrogen Boride Nanosheets**

Miwa Hikichi, Jumpei Takeshita, Junyan Han, Shin-ichi Ito, Osamu Oki, Ryuki Tsuji, Akira Hasegawa, Samuel Jeong, Yoshikazu Ito, Iwao Matsuda, Hayato Tsurugi, Masahiro Miyauchi, and Takahiro Kondo*

**This file includes:**

**Figure S1.** Thermogravimetric curves

**Figure S2.** TG curves and temperature programmed desorption (TPD) of m/z = 28.

**Figure S3.** Typical TG and TPD results

**Figure S4.** Differential thermal analysis curves.

**Figure S5.** X-ray photoelectron spectroscopy (XPS).

**Figure S6.** X-ray diffraction patterns.

**Figure S7**. Surface area analysis of pure HB and Pyrazine-HB using the Brunauer-Emmett-Teller method.

**Figure S8.** Transmission electron microscopy (TEM) images and line profiles.

**Figure S9.** Scanning transmission electron microscopy (STEM) images and EDS mapping of boron and nitrogen.

**Figure. S10**. Emission spectra of UV and xenon lamps used for photoinduced hydrogen evolution measurements

**Table S1.** Cartesian coordinates of pyrazine.


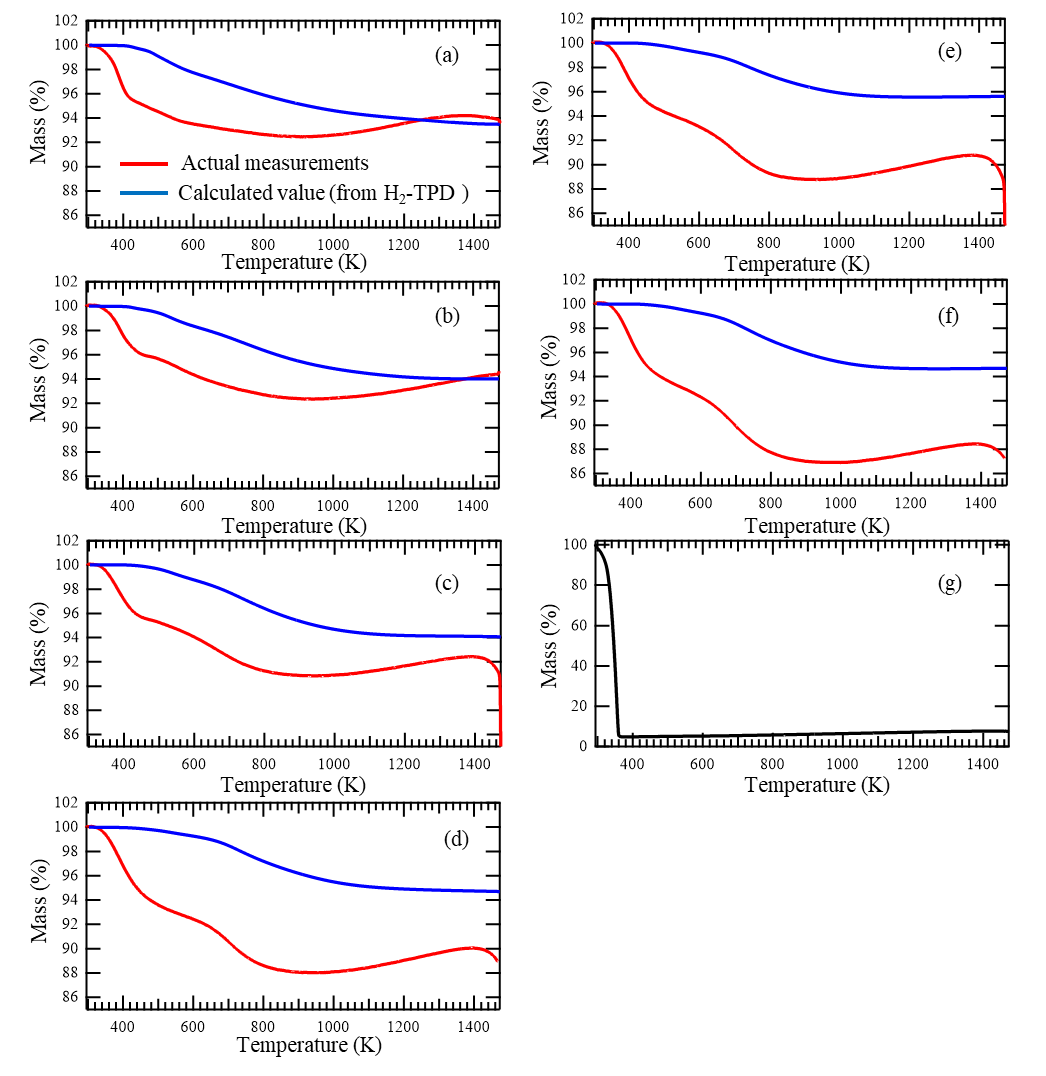


**Figure S1.** Thermogravimetric (TG) curves. TG curves of (a) pristine HB, (b) Pyrazine (0.50 mol%)-HB (initial feed ratio HB:Pyrazine = 1:0.01), (c) Pyrazine (2.5 mol%)-HB (1:0.05), (d) Pyrazine (2.9 mol%)-HB (1:0.5), (e) Pyrazine (3.3 mol%)-HB (1:0.1), (f) Pyrazine (3.8 mol%)-HB (1:1), and (g) pure pyrazine. The red curves represent the experimentally measured TG profiles, and the blue curves correspond to the calculated mass loss attributed solely to hydrogen desorption based on H_2_ evolution. The TG curves of the Pyrazine-HB samples show greater overall weight loss, which cannot be explained by hydrogen release alone, suggesting the presence and subsequent desorption of intercalated or weakly bound pyrazine molecules.


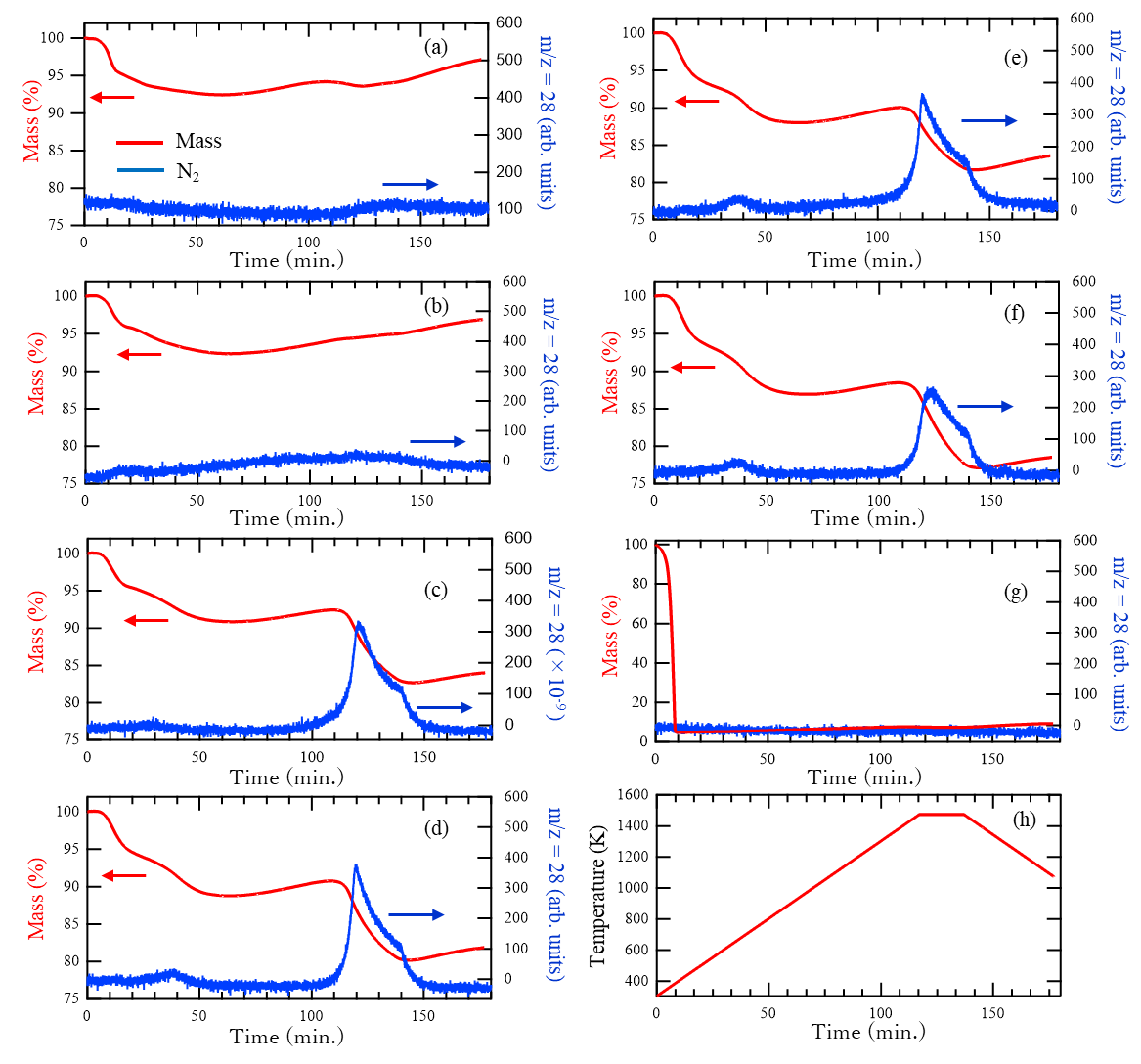


**Figure S2.** TG curves and temperature programmed desorption (TPD) of m/z = 28. TG curves of (a) pristine HB, (b) Pyrazine-HB (initial feed ratio HB:Pyrazine = 1:0.01), (c) Pyrazine-HB (1:0.05), (d) Pyrazine-HB (1:0.1), (e) Pyrazine-HB (1:0.5), (f) Pyrazine-HB (1:1), and (g) pure pyrazine. (h) Temperature profile against time for TG measurements. The red curves represent the experimentally measured TG profiles, and the blue curves correspond to the m/z = 28 intensity in TPD.


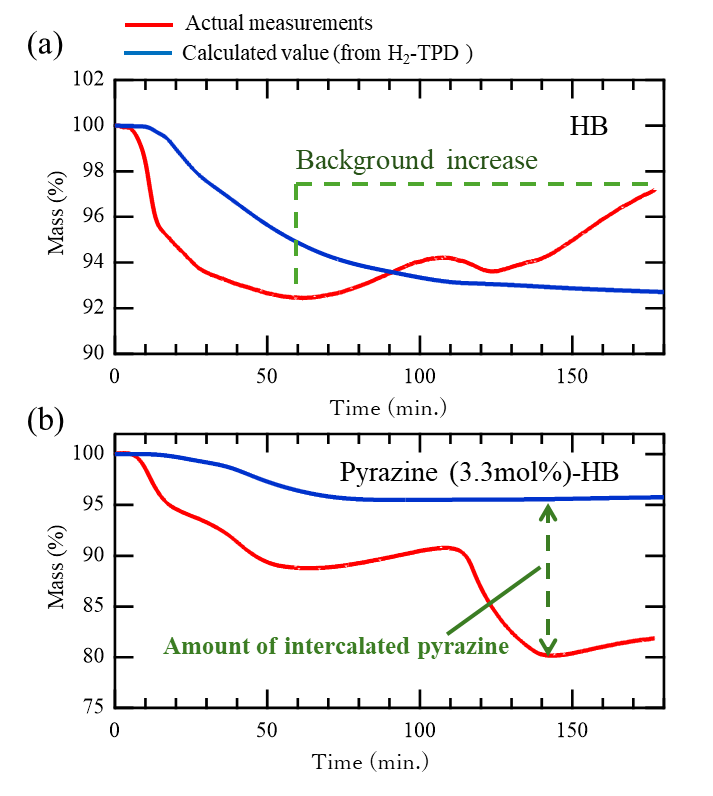


**Figure S3.** Typical TG and TPD results. Representative TG and TPD results (same as Figure S1 and Figure S2) for (a) HB and (b) Pyrazine (3.3mol%)-HB.


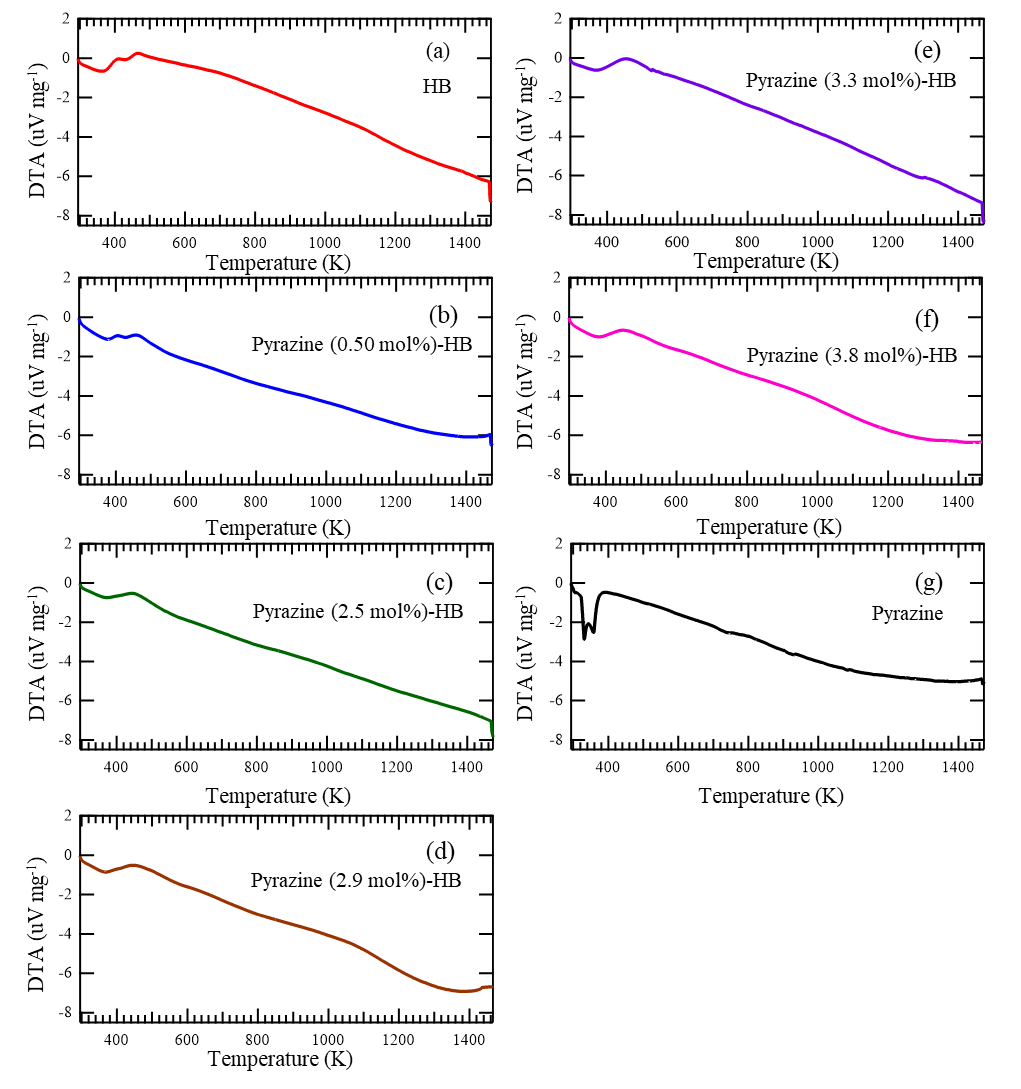


**Figure S4.** Differential thermal analysis (DTA) curves. DTA curves of (a) pristine HB, (b) Pyrazine (0.50 mol%)-HB, (c) Pyrazine (2.5 mol%)-HB, (d) Pyrazine (2.9 mol%)-HB, (e) Pyrazine (3.3 mol%)-HB, (f) Pyrazine (3.8 mol%)-HB, and (g) pure pyrazine.


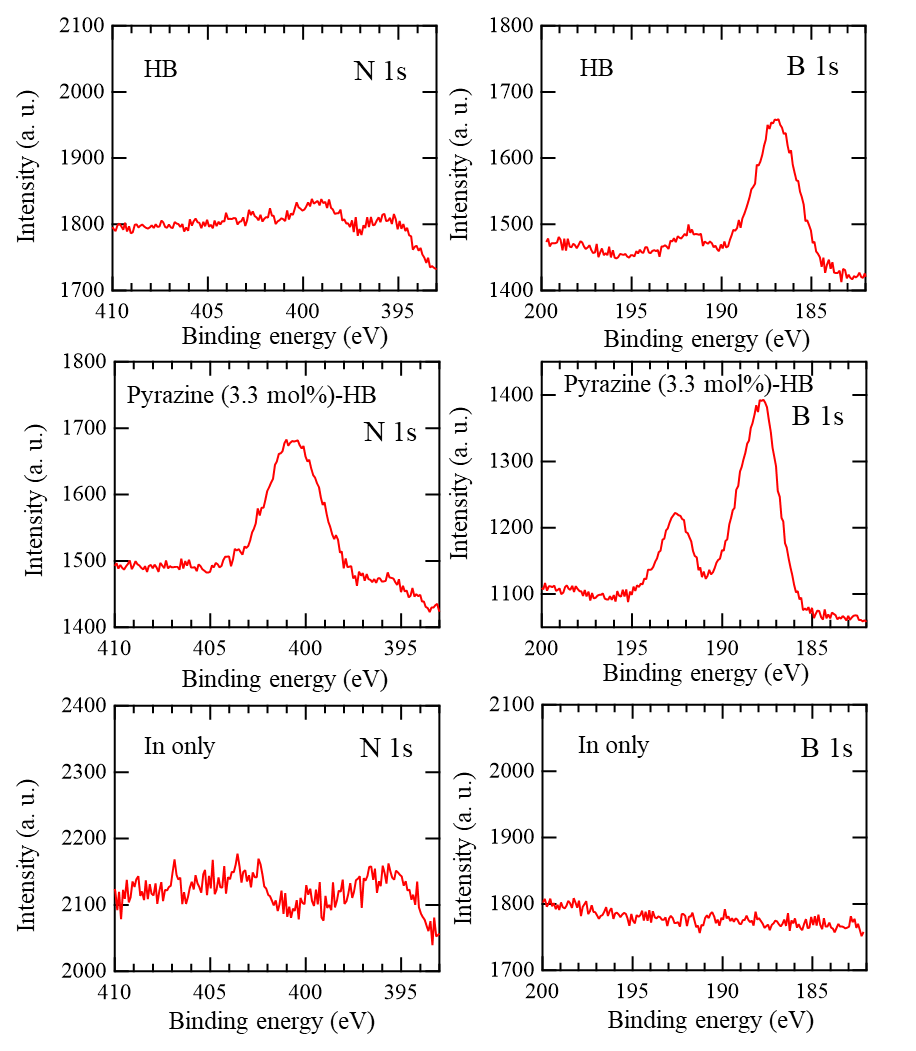


**Figure S5.** X-ray photoelectron spectroscopy (XPS) profiles. XPS profiles of N 1s and B 1s for pristine HB on In, Pyrazine (3.3 mol%)-HB on In, and the In substrate.


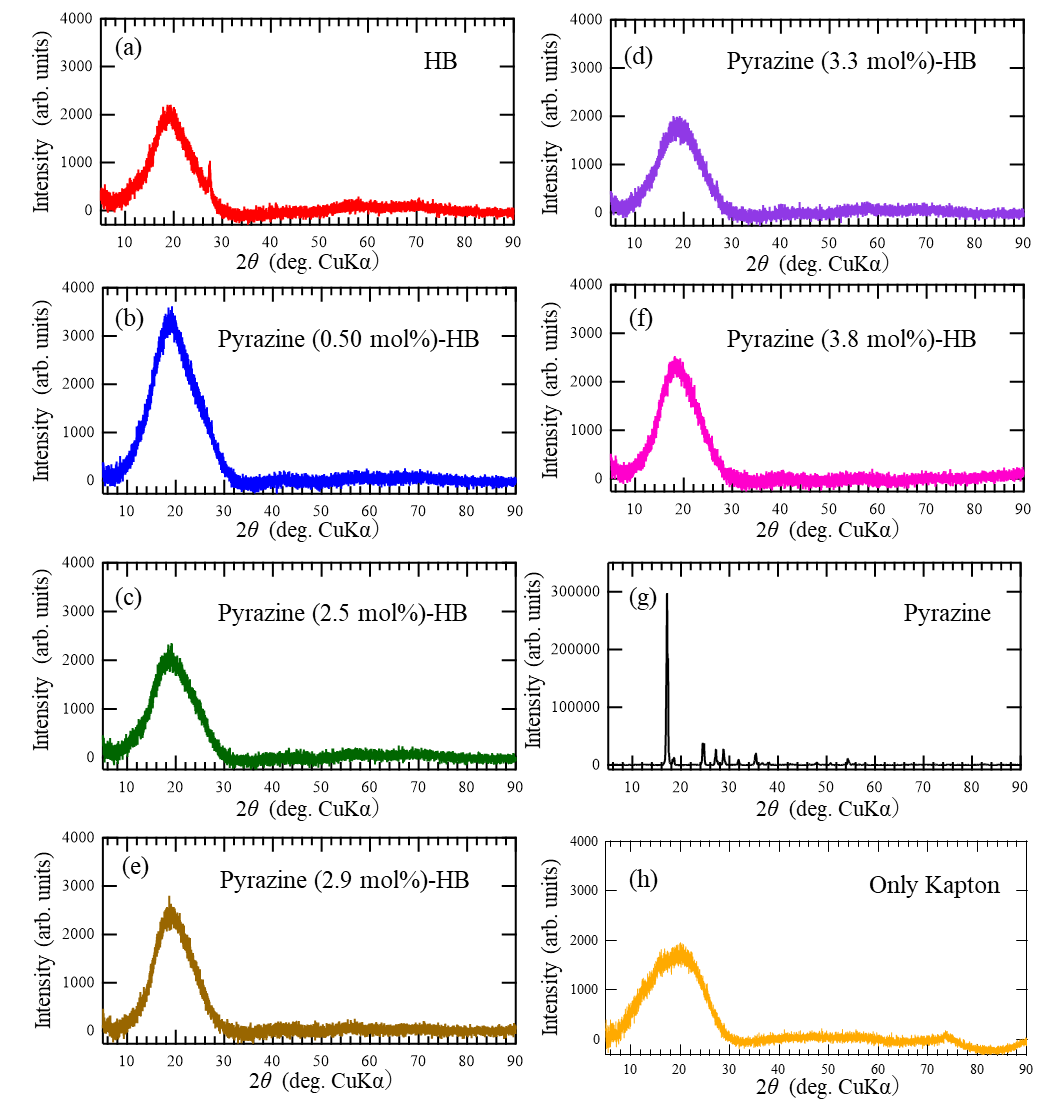


**Figure S6.** X-ray diffraction (XRD) patterns. XRD patterns of (a) pristine HB, (b) Pyrazine (0.50 mol%)-HB, (c) Pyrazine (2.5 mol%)-HB, (d) Pyrazine (2.9 mol%)-HB, (e) Pyrazine (3.3 mol%)-HB, and (f) Pyrazine (3.8 mol%)-HB measured under Ar atmosphere using Kapton® capsules. XRD patterns of (g) crystalline pyrazine reference and (h) empty Kapton capsule as background reference. The broad feature centered near 2θ = 20° originates from the Kapton film used for sample encapsulation. No sharp diffraction peaks were observed in Pyrazine-HB samples (b–f), indicating the absence of long-range order and suggesting uniform dispersion or intercalation of pyrazine within the HB nanosheet matrix.


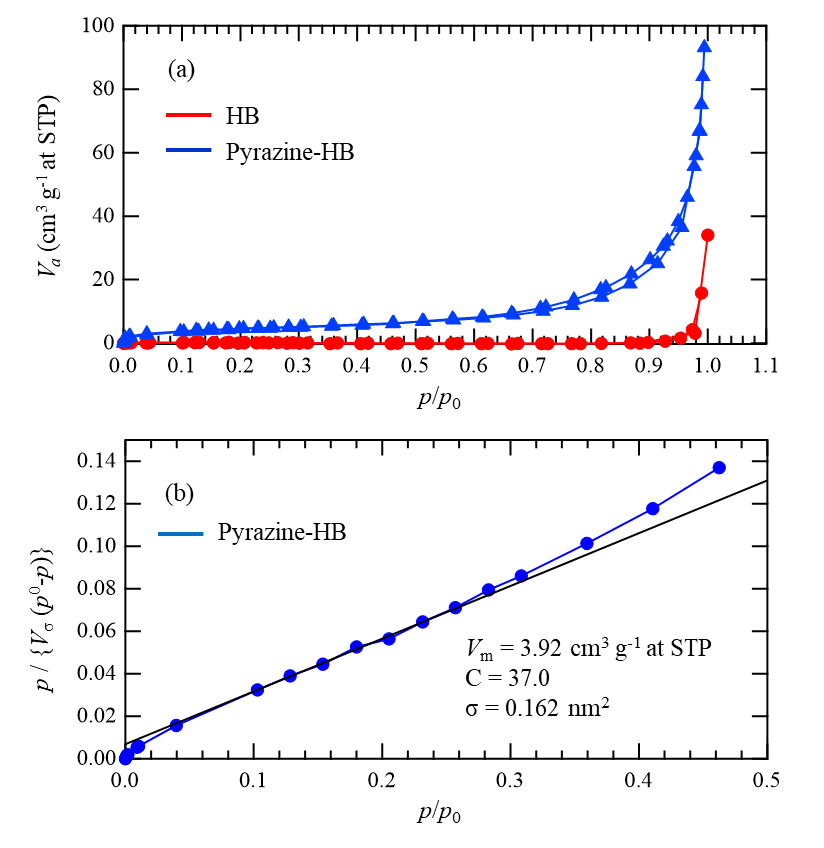


**Figure S7**. Surface area analysis of pristine HB and Pyrazine-HB using the Brunauer-Emmett-Teller (BET) method. (a) Nitrogen adsorption–desorption isotherms of pristine HB and Pyrazine-HB (3.3 mol%). Pristine HB shows negligible adsorption, suggesting aggregation and restacking of the nanosheets. In contrast, Pyrazine-HB displays nitrogen uptake, indicating improved surface accessibility due to pyrazine intercalation. (b) BET plot of Pyrazine-HB (3.3 mol%), where *V*_m_ is the adsorbed quantity, *p* is the equilibrium adsorption pressure, *p*_0_ is the saturated vapor pressure, *V*_m_ is the monolayer adsorption capacity, *C* is a BET constant, and σ is the cross-sectional area of nitrogen on the surface. BET plot yields a calculated specific surface area of 17 m^2^ g^-1^.


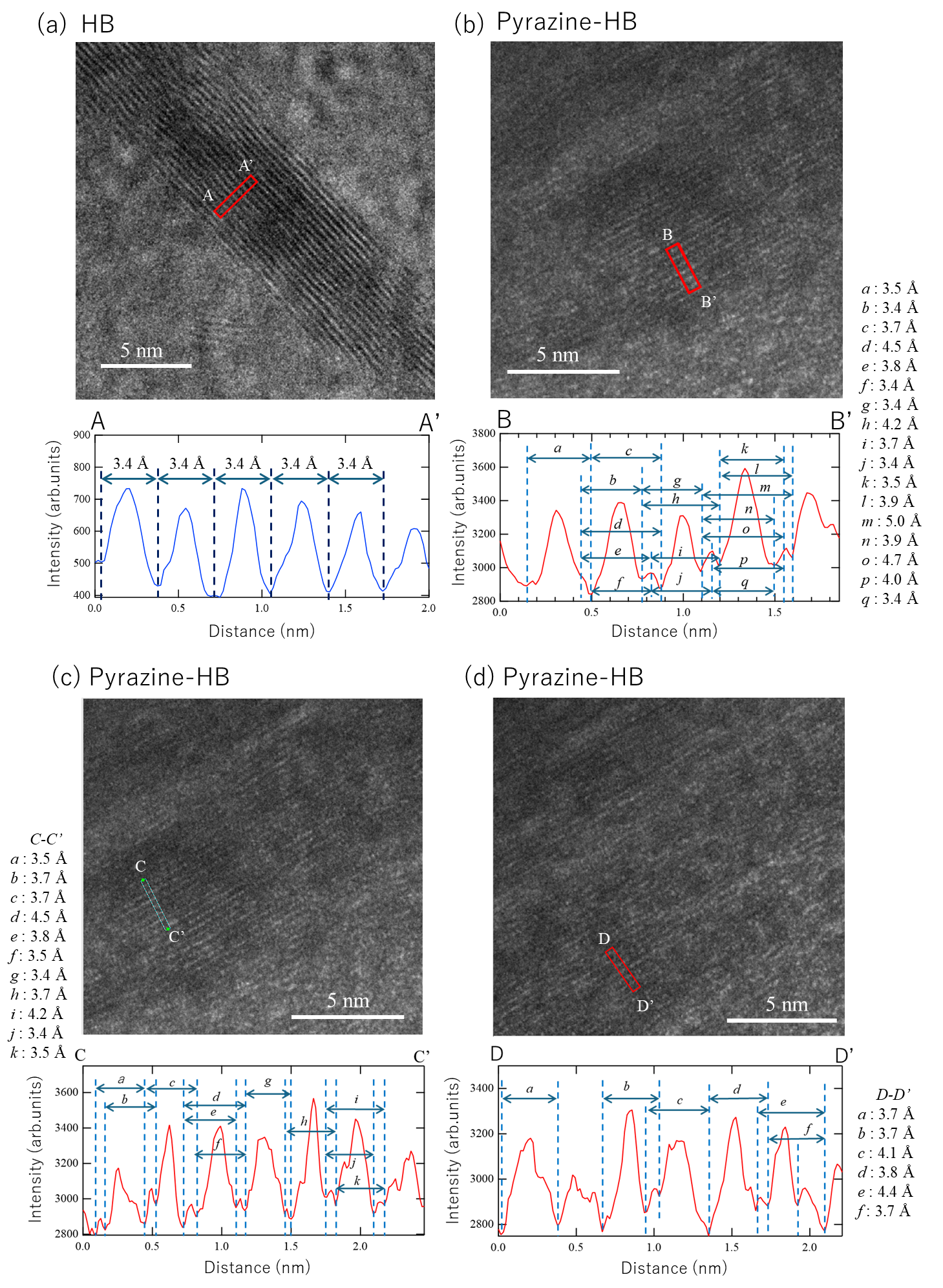


**Figure S8**. Transmission electron microscopy (TEM) images and line profiles. TEM images and corresponding line profiles for (a) pristine HB and (b) Pyrazine (3.3 mol%)-HB. Distances of line profiles in B-B‘, C-C‘, and D-D‘ are shown together.


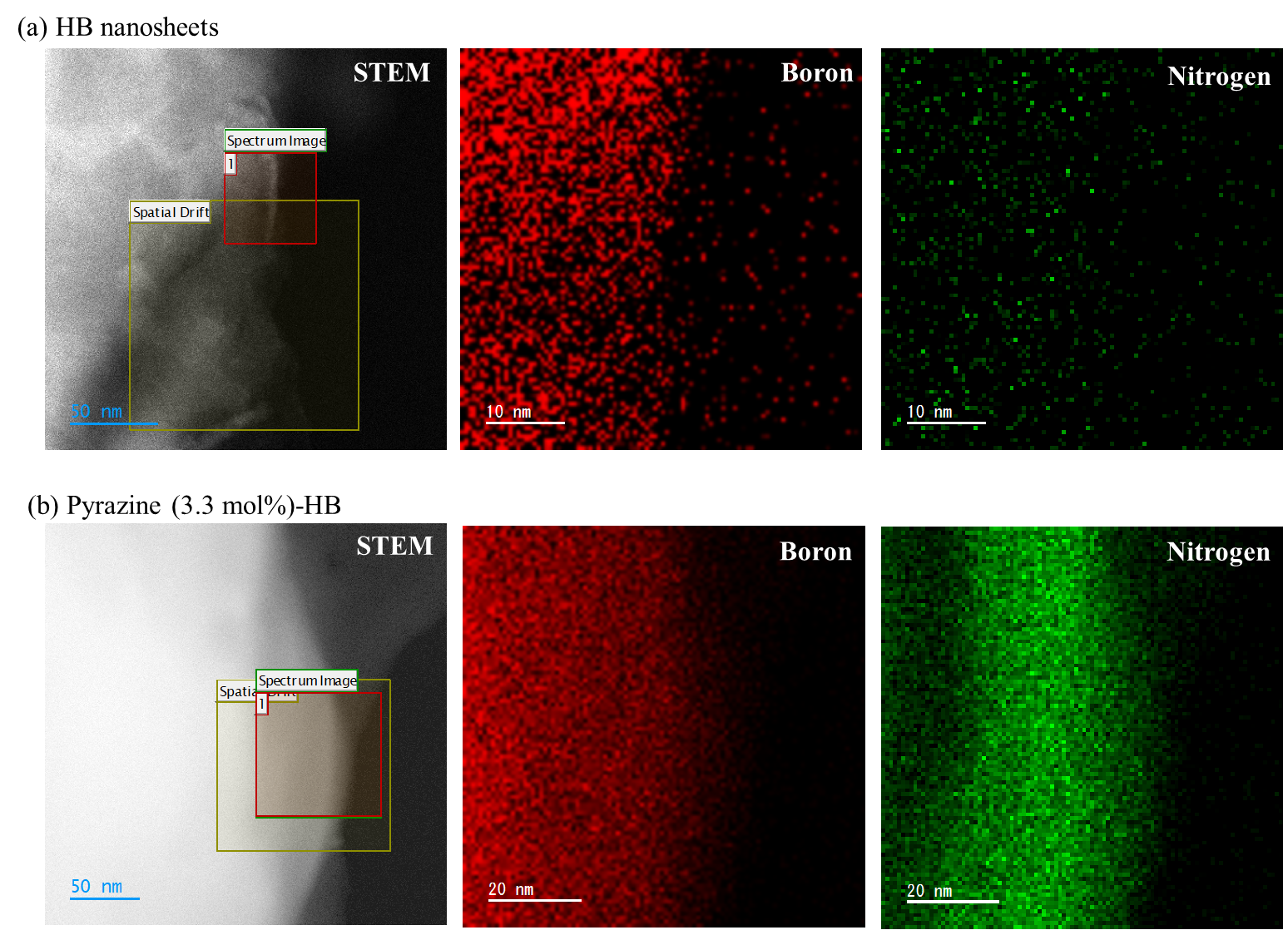


**Figure S9**. Scanning transmission electron microscopy (STEM) images and EDS mapping. STEM and elemental maps of boron and nitrogen for (a) pristine HB and (b) Pyrazine (3.3 mol%)-HB.


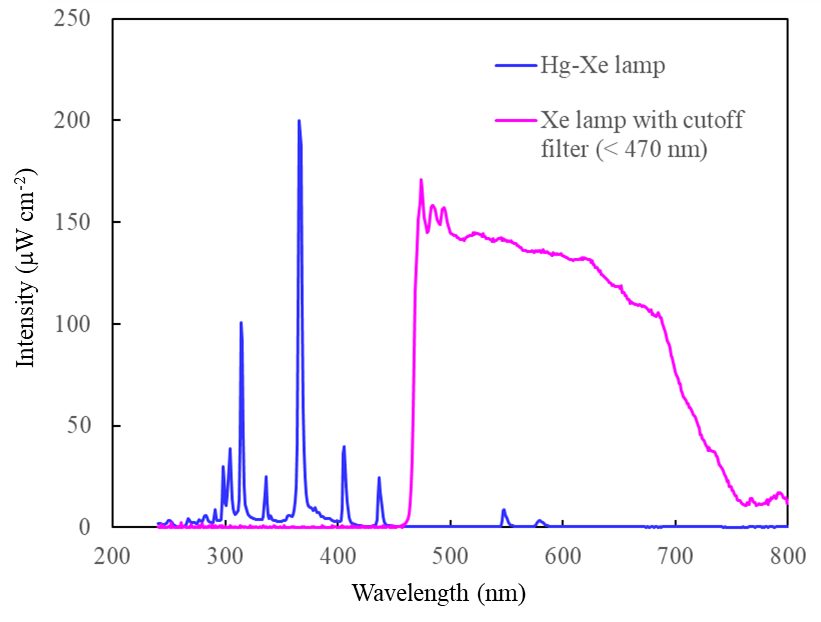


**Figure. S10**. Emission spectra of UV and xenon lamps used for photoinduced hydrogen evolution measurements. Wavelength spectra of UV irradiation was performed with a 150 W Hg–Xe lamp, and visible light irradiation was performed with a 500 W Xe lamp equipped with a UV short wavelength cutoff filter (λ < 400 nm, 470 nm cutoff).

**Table S1.** Cartesian coordinates of pyrazine.

C -0.00585 0.08637 -0.00000

C 1.38875 0.08638 0.00001

N 2.09540 1.21858 -0.00001

C 1.38873 2.35076 -0.00005

C -0.00588 2.35075 -0.00007

N -0.71253 1.21855 -0.00004

H -0.56294 -0.84532 0.00002

H 1.94586 -0.84529 0.00005

H 1.94582 3.28245 -0.00008

H -0.56298 3.28243 -0.00010
